# Impact of structured wheelchair services on satisfaction and function of wheelchair users in Zimbabwe

**DOI:** 10.4102/ajod.v5i1.222

**Published:** 2016-06-10

**Authors:** Surona Visagie, Tecla Mlambo, Judith van der Veen, Clement Nhunzvi, Deborah Tigere, Elsje Scheffler

**Affiliations:** 1 Centre for Rehabilitation Studies, Stellenbosch University, South Africa; 2 Department of Rehabilitation, College of Health Sciences; University of Zimbabwe, Zimbabwe; 3 Christian Blind Mission Regional Office, South Africa

## Abstract

**Background:**

Providing wheelchairs without comprehensive support services might be detrimental to user satisfaction and function.

**Objectives:**

This paper compares wheelchair user satisfaction and function before and after implementation of comprehensive wheelchair services, based on the World Health Organization guidelines on wheelchair service provision in less resourced settings, in Zimbabwe.

**Method:**

A pre- and post-test study with a qualitative component was done. Quantitative data were collected with the Quebec User Evaluation of Satisfaction with Assistive Technology for adults and children and the ‘Functioning Every day with a Wheelchair Questionnaire’. Data were collected from 55 consecutively sampled wheelchair users, who received a new wheelchair in the study period. Qualitative data were collected through two audio recorded focus groups and two case studies and are presented through narrative examples.

**Results:**

The proportion of adult users who were satisfied significantly increased for all wheelchair and service delivery aspects (*p* = 0.001 - 0.008), except follow-up (*p* = 0.128). The same was true for children’s post-test ratings on all variables assessed (*p* = 0.001 - 0.04), except training in the use of the device (*p* = 0.052). The biggest improvement in satisfaction figures were for comfort needs (44.3%), indoor mobility (43.2%), outdoor mobility (37.2%), safe and efficient, independent operation (33.5%) and transport (31.4%). The qualitative data illustrated user satisfaction with wheelchair features and services.

**Conclusion:**

The wheelchair service programme resulted in significant positive changes in user satisfaction with the wheelchair, wheelchair services and function. It is recommended that the Zimbabwean government and partner organisations continue to support and develop wheelchair services along these guidelines.

## Introduction

Wheelchairs are often essential assistive devices for persons with mobility limitations. However, wheelchairs, like shoes, are available in different designs and sizes in order to meet different functional, posture support and environmental needs (Borg, Lindstrom & Larsson 2011; Pearlman *et al.* 2008; WHO 2008). An ill-fitting or inappropriate wheelchair which fails to meet the user’s posture support, functional and/or environmental needs leads to dissatisfaction, which may result in sub-optimal use or abandonment of these expensive devices (Mukherjee & Samanta 2005; Toro *et al.* 2012). Therefore assessment, prescription of an appropriate wheelchair, fitting, user training, maintenance and follow-up are as essential to wheelchair provision as the wheelchair itself (Bergstrom & Samuelson 2006; De Groot *et al.* 2011; Glumac *et al.* 2009; Hansen, Tresse & Gunnarsson 2004; Routhier *et al.* 2003; Samuelsson & Wressle 2008; Toro *et al.* 2012; WHO 2008).

Wheelchair provision in Southern Africa and Zimbabwe are often dependent on donations. Unfortunately, in the past, many donor organisations have provided wheelchairs with no or little accompanying services. Often, these donated wheelchairs were inappropriate for the user’s needs (Øderud 2014; Visagie *et al.* 2015). Visagie *et al.* (2015) found that users in Zimbabwe were much less satisfied with their wheelchairs, wheelchair services and function in their wheelchairs than wheelchair users in resourced settings.

The World Health Organization published guidelines on wheelchair provision in less resourced settings (WHO wheelchair guidelines) (WHO 2008). While studies reporting on the implementation of and/or adherence to these guidelines could be identified (Borg *et al.* 2011; Visagie, Scheffler & Schneider 2013), no evidence of the impact of these guidelines on service delivery could be found. Greer, Brasure and Wilt (2012) advocate for the development of an evidence base for wheelchair services focusing on users, interventions, comparisons and outcomes. This paper aims to contribute to this evidence base by comparing users’ satisfaction and function with their wheelchairs before and after implementation of comprehensive wheelchair services based on the WHO wheelchair guidelines.

## The comprehensive mobility support project

In 2012 the Comprehensive Mobility Support Project (CMSP) was implemented in Zimbabwe by the Jairosi Jiri Association (JJA) in partnership with Christian Blind Mission (CBM) and the Zimbabwean Ministry of Health and Child Care (MOHCC) with financial support from United States Agency for International Development (USAID). The aim was to improve and professionalise wheelchair service delivery. The project was implemented in six Zimbabwean provinces over 37 months (January 2012 to February 2015). By adopting the WHO wheelchair guidelines and the eight services steps presented in Table 1 (WHO 2008:76) as service delivery model, the CMSP followed a user-centred and rights-based approach.

**TABLE 1:** World Health Organization guidelines for wheelchair provision in a less resourced setting.

Service step	Objective	Good practice
Referral and appointment	To ensure equitable access to services.	Open file and make appointment. Train referral network personnel.
Assessment	Assess the need of each user accurately to prescribe the most appropriate wheelchair.	Individualised assessment by trained personnel. Assessment equipment readily available. Taking into account the user’s physical condition, environment, lifestyle, age, gender and culture. Findings are documented and filed.
Prescription	Match the needs of the user with the most suitable wheelchair.	Service personnel and users together select the final wheelchair and necessary features. User tries out various options. Prescription is fully documented.
Funding and ordering	Order and procure the prescribed wheelchair as soon as possible.	Clear ordering and procurement systems. Stock decreases delivery times. Agreements with suppliers on ordering and delivering. Inform user of expected waiting times.
Product preparation	Prepare wheelchair for fitting.	Trained providers set up wheelchair according to user needs, including modifications and installation of custom or posture support devices. Do safety and readiness check before fitting.
Fitting	Assemble wheelchair correctly and make adjustments to ensure optimal fit.	Trained providers do fitting and make the required adjustments. Fit is done with user only sitting in the wheelchair and pushing the wheelchair. Additional fitting appointments may be needed for users with more complex needs.
Training	Provide users and caregivers with information and training needed to use wheelchair effectively and safely.	Trained providers and/or peer trainers. Users trained in: Mobility in wheelchair Safety Transfers Basic repairs and maintenance
Follow-up, maintenance and repair	Maximise function, comfort and safety during follow-up and ensure appropriate maintenance of wheelchair.	Follow-up appointments made. Frequency determined by need. Prioritise children, users at risk for pressure sores or postural deviations and those using posture support devices. Includes clinical, technical and training aspects. Gather feedback on services.

*Source*: WHO 2008

### Outcomes of the comprehensive mobility support project

Sixteen seating clinics were established in six provinces in Zimbabwe.

A total of 59 rehabilitation service providers^1^ were trained in basic wheelchair service delivery and 30 in intermediate wheelchair service delivery using the WHO basic and intermediate level wheelchair service training packages^2^ (WSTP-B and WSTP-I). The training was complemented with follow-up workshops, clinical mentoring and support, and peer support sessions.

Fifteen of the service providers attended a training of trainer’s programme as a first step towards them qualifying as trainers to provide the WSTP-B in the future.

Twenty wheelchair workshop personnel^3^ were trained in wheelchair assembly, fabrication, modification, maintenance and repairs.

Tools, spares, consumables and materials to modify wheelchairs were provided to the six wheelchair workshops supporting the 16 seating clinics.

A total of 1316 wheelchairs were procured, distributed between seating clinics and issued to users. Rehabilitation service personnel conducted assessments, prescriptions, user training and follow-up. Wheelchair technicians assisted with user fitting, made wheelchair modifications, fabricated low-cost posture support devices, refurbished second-hand wheelchairs, and offered repair and maintenance services.

### Design of wheelchairs issued

**FIGURE 1:**
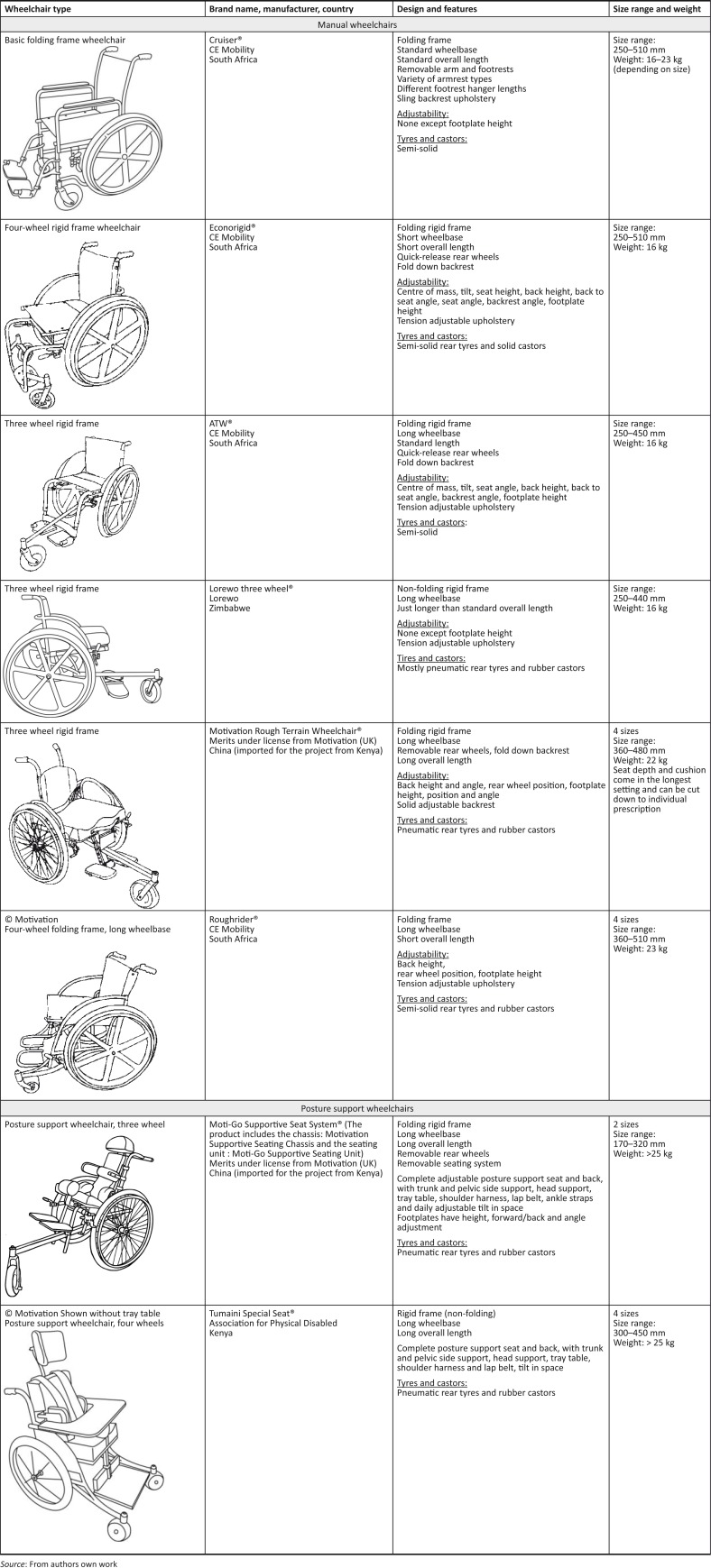
Design and features of wheelchairs issued to study participants.

Users primarily used basic folding or non-folding, four-wheel frame wheelchairs when they accessed the clinics for a new wheelchair as described by Visagie *et al.* (2015). Users were prescribed and fitted with one of the wheelchair designs listed and described in Figure 1. These wheelchairs were manufactured in Zimbabwe, or imported from Kenya and South Africa. All these wheelchairs, except the basic folding frame and the compact urban-use wheelchairs, had long wheelbases. All wheelchairs, except the basic folding frame and locally manufactured Lorewo wheelchairs, had adjustable settings to optimise fit, posture support, function and propulsion. All wheelchair frames were manufactured from mild steel. The majority of Lorewo wheelchairs, the Association for the Physically Disabled, Kenya (APDK) and the Motivation Products had pneumatic rear tyres while the South African products all had semi-solid tyres.

Wheelchair users presented with a range of posture support, functional and environmental needs. They were provided with the most appropriate wheelchair available and, where needed, locally made posture support devices were fitted to manual wheelchairs. The posture support wheelchairs were mostly reserved for children who needed all the posture support options of these wheelchairs.

## Method

A mixed method descriptive study design with a pre- and post-test component was done. Qualitative data were collected to explore and contextualise quantitative findings through the experiences and perceptions of individual participants (Kroll, Neri & Miller 2005).

### Quantitative phase

Persons who accessed the 16 seating clinics where the CMSP was implemented, between 31 October 2013 and 28 February 2014, for a manual wheelchair, were consecutively sampled to participate in the study (*n* = 135). For this pre-test post-test component all who were not previous wheelchair users or did not get a new wheelchair in the study period were excluded. This resulted in 55 participants.

Quantitative data were collected through a self-designed demographic questionnaire and three standardised tools, that is, the Quebec User Evaluation of Satisfaction with Assistive Technology (QUEST 2.0) for adults (Demers *et al.* 2002), the QUEST 2.1 for children (Murchland, Kernot & Parkyn 2011) and Functioning Everyday with a Wheelchair Questionnaire (FEW) (Mills, Holm & Schmeler 2007; Mills *et al.* 2002). The QUEST 2.0 and QUEST 2.1 assess user satisfaction with assistive devices and service provision processes. Both tools were found valid and reliable in Global North settings (Demers *et al* 2002; Murchland *et al.* 2011). The FEW assesses users’ perceptions of the impact of the wheelchair on their function through 10 items. The FEW has been found to capture 96.9%–99.7% of user’s goals in wheelchair use with moderate precision for test–retest reliability (Mills *et al.* 2007). The tools were translated into Shona and Ndebele. The forward translations were done by two occupational therapists who were native Shona and Ndebele speakers. A multi-linguist from the Medical Research Council of Zimbabwe reviewed and compared both translations to the original English versions for correctness and consistency.

Pre-test data were collected when users first accessed the service (31 October 2013 to 28 February 2014). Post-test data were collected between 3 and 5 months after they received a new wheelchair (1 March 2014 to 30 May 2014). Data were coded and entered into Microsoft Excel before being imported into Stata 13.0 for analysis. A two-sample test of proportions was carried out to determine whether there was a statistically significant difference in the percentage of satisfied participants between the pre- and post-test ratings. An alpha level of 0.05 was selected.

### Qualitative phase

The study population for this phase included all users, their family members and/or caregivers who accessed the 16 seating clinics as well as service providers at these clinics. Through purposive sampling, 22 participants were identified to participate in two focus group discussions. A further two participants (a boy aged 9 and a woman aged 26) were identified in the same manner to participate in two case studies.

The focus group discussions lasted 4 hours each. They were held in a rural setting of Masvingo province (January 2014) and in an urban setting of Harare province (April 2014). A focus group discussion guide was used. Topics explored included:

- Participants’ experiences and problems in life situations.
- Satisfaction with their wheelchairs.
- How the experience of wheelchair users in Zimbabwe can be improved.

The focus group discussions and case study interviews were audio recorded and transcribed verbatim. Findings relevant to this paper are included as narrative examples under the relevant quantitative sections.

### Ethical considerations

Ethical approval was granted by the Joint Research Ethics Committee (JREC/323/13) of the University of Zimbabwe, College of Health Sciences, and the Medical Research Council of Zimbabwe (MRCZ/A/1813). Written informed consent was obtained from all wheelchair users, parents, guardians and caregivers as appropriate, as well as assent for child participants. Parents, guardians and/or caregivers became proxy respondents for participants who were not able to communicate or understand and answer the questions on their own because of the nature of their disabilities. Participation was voluntary; participants could withdraw from the study without adverse consequences to them, and participant privacy and confidentiality were maintained.

## Results

### Demographic information

The median age of the 55 study participants was 21 years (interquartile range 11–43). There were 29 (53%) adults and 26 (47%) children. The median age of the adults was 42.5 years with an interquartile range of 26.5–62, while that of the children was 11 years with an interquartile range 7–13. The majority (62%) were male and 38% were female. Of the adults, one was formally employed and 11 were informally employed. Sixteen (64%) of the 25 children whose school attendance was recorded were attending school. Slightly more participants lived in urban areas (52.7%) than in rural (40%) areas, while 1.8% lived in peri-urban areas and 5.5% indicated they had to function in both urban and rural areas. The majority of participants used public transport (86.6%).

The most common diagnosis was cerebral palsy (43.6%), followed by spinal cord injury/paraplegia (18.2%), polio myelitis (9%) and muscular dystrophy (5.4%).

Wheelchair services were mostly provided by rehabilitation technicians (62.5%) (Table 2). Therapists provided wheelchair services to 7.5% of participants.

**TABLE 2:** Wheelchair service providers (*n* = 40) (15 participants could not answer this question).

Provider	Number of providers	Percentage of participants
Physiotherapist	3	7.5
Rehabilitation technicians	25	62.5
Orthopaedic technician	1	2.5
Wheelchair technician	4	10.0
Other	7	17.5

*Source*: From authors own work

### Satisfaction of adult participants with wheelchair features and services

QUEST 2.0 manual outlines that items in which between 25% and 33% or more users are ‘somewhat satisfied’, ‘dissatisfied’ or ‘very dissatisfied’ require attention (Demers, Weiss-Lambrou & Ska 2000). Accordingly, the five-point scale of the QUEST 2.0 was collapsed into two categories (‘quite’ or ‘very satisfied’ in one category and somewhat satisfied’, ‘dissatisfied’ or ‘very dissatisfied’ in the other category). Adult wheelchair users’ pre- and post-test QUEST 2.0 ratings are presented in Table 3.

**TABLE 3:** Comparison of adults’ satisfaction ratings (QUEST 2.0) with wheelchair features and wheelchair service delivery before and after implementation of CMSP (*n* = 29).

Wheelchair features/services	Quite or very satisfied			

	Pre-test	Post-test	95% CI of the differences between pre- and post-satisfaction	*p*
**Wheelchair features**				
Dimensions	39.3	90	-72.0 to -30.0	0.001
Weight	52	93.4	-61.9 to -20.1	0.001
Ease of adjustment	43	83	-62.9 to -17.1	0.002
Safety	39.3	80	-64.1 to -18.0	0.001
Durability	53.5	89.7	-57.4 to -14.6	0.002
Ease to use	32.2	96.7	-83.3 to -46.7	0.001
Comfort	26	96.7	-88.6 to -53.4	0.001
Effectiveness	35.7	89.6	-74.9 to -33.1	0.001
**Wheelchair services**				
Service delivery	42.8	86.7	-65.9 to -22.1	0.001
Repairs and servicing	32	89.3	-77.8 to -36.2	0.001
Professional service	64.3	93	-49.1 to -8.9	0.008
Follow-up	57	76	-43.0 to -5.04	0.128

*Source*: From authors own work

Pre-test ratings illustrate low levels of satisfaction (maximum 64.3%) with all wheelchair features and all wheelchair service aspects. In contrast, post-test ratings indicated high levels of satisfaction with 76% or more users satisfied with every aspect of their wheelchair and service delivery.

The improvement in the proportion of participants satisfied with wheelchair features was statistically significant for all items (*p* ≤ 0.002) (Table 3). The qualitative data further illustrate user satisfaction with wheelchair features. One of the case study participants described her new wheelchair as ‘a chair made for me’. She continued: ‘I am very satisfied with my wheelchair because they have given me exactly the right size’ (Female, 26, user).

Qualitative data showed that users and providers concurred on the importance of appropriate wheelchair features to enhance safety, function and mobility:

‘… the right size with all safety features is important to me…I think it’s because I used to fall a lot (with previous wheelchair)…’ (Male, 25, user)

‘…when I am safe I move faster and I am confident to do it…’ (Male, 44, user and provider)

According to focus group participants, non-folding wheelchairs and the bulkiness of the folding rigid frame^4^ design of some of the wheelchairs limited transport options. Users preferred folding wheelchairs for easy transportation although they recognised the durability limitations of the basic folding frame wheelchairs with active use in harsh environments:

‘…foldable ones are not durable but they work best when it comes to transportation …I think it’s a 50-50 situation…’ (Male, 44, user and provider)

‘… you cannot take it everywhere… it’s not foldable and that’s my problem with this one…’ (Female, 33, user)

As with wheelchair features, the improvement in satisfaction with wheelchair services was also statistically significant for all items (*p* ≤ 0.008) except for follow-up (*p* = 0.128) (Table 3). Users expressed satisfaction with having a dedicated service offering professional services, as well as the service delivery procedures and the length of time it took to receive their wheelchairs. They were concerned about losing this level of professional support:

‘…our greatest fear is that you may dump us and we will not be getting continued support…’ (Male, 27, user)

‘It is much better when you know where to go and be helped on time. It worked well for me…’ (Male, 41, user)

‘…they followed the dates they had told me…they were nice to me and the wheelchair is working well…’ (Female, 26, user)

Having wheelchairs available at the clinic level improved service delivery by not only reducing waiting time but also by offering users the opportunity to trial chairs and experience the different features and designs. Users were therefore directly involved and engaged with providers on their wheelchair prescription, thus further enhancing a user-centred approach:

‘…waiting period was short because we had the chairs at the clinic…’ (Male, 33, provider)

‘When I got it, there were many chairs and I had to try them one by one until I got the right one.’ (Male, 27, user)

‘…to provide the best, we need to present options and also hear from the user or caregiver about their surroundings… and they will get the right chair…’ (Male, 33, provider)

Training and information on wheelchair features and functions as well as basic maintenance were provided:

‘We are doing it better and they seem happy, I think it’s because of the training…’ (Female, 29, provider)

The provision of service kits might have helped participants to maintain their own wheelchairs:

‘… it’s true, service kits will help us service our chairs rather than wait for the rehab centres to do it for us…’ (Male, 41, user)

A service provider raised concerns about providing follow-up in rural areas:

‘Some of them we cannot follow them up, because they live too far from our centres and their areas are not easily accessible by road even in the few instances we get transport.’ (Male, 30, service provider)

### Satisfaction of child participants with wheelchair features and services

Similar to the adults, pre-test QUEST 2.1 ratings illustrate high levels of dissatisfaction with all wheelchair features and all but one of the wheelchair service aspects. Post-test ratings illustrated significant improvement in satisfaction levels with between 79.2% and 100% of users satisfied with the various items (Table 4).

**TABLE 4:** Comparison of child QUEST 2.1 satisfaction ratings of child users with wheelchair features and wheelchair service delivery before and after implementation of CMSP (*n* = 26).

Wheelchair features/services	Quite or very satisfied			

	Pre-test	Post-test	95% CI	*p*
**Wheelchair design features**				
Size	55.6	88.0	-54.6 to -9.4	0.011
Weight	63.0	96.0	-52.8 to -13.2	0.004
Ease to push	63.0	87.5	-47.4 to -2.6	0.040
Aesthetics	55.6	100	-62.7 to -25.3	0.001
Ease to use	59.3	95.8	-57.1 to -16.9	0.002
Set up time	45.3	100	-84.4 to -25.6	0.001
Reliability	32.2	95.8	-90.5 to -37.5	0.001
Meeting needs	46.0	91.7	-75.2 to -16.8	0.002
**Wheelchair services**				
Advice on chair selection	60.0	95.8	-67.0 to -9.0	0.004
Waiting time	40.0	87.5	-71.2 to -24.8	0.001
Repairs and servicing	40.0	79.2	-64.2 to -13.8	0.006
Training in use	77.0	95.8	-37.0 to -1.0	0.052

*Source*: From authors own work

All changes in satisfaction with wheelchair features and services were statistically significant (*p* ≤ 0.04), except for training in the use of the device, which showed acceptable satisfaction levels in the pre-test (77%) (Table 4). The impact of appropriate wheelchair features on function, posture support, safety and comfort is illustrated by user feedback:

‘…I am safer and comfortable in this one… it’s the right size, I like it and its beautiful too…’ (Female, 11, user)

‘… it’s not giving me problems, it’s not making me fall…’ (Male, 9, user)

‘I have seen a great improvement especially that she can now sit upright in her chair… I am happy for now…’ (Female, 39, caregiver)

Similar to adults, users and caregivers expressed satisfaction with services and being included in the decision-making process, but were concerned about future services.

‘…we were asked for our input, like what we preferred on this one. I am happy he is using it…he goes out to play with others…I think its light and it’s the right size for him…’ (Female, 47, caregiver)

‘If [user’s name] outgrows the current wheelchair, is the programme going to help him get another one?’ (Female, 47, caregiver)

Pneumatic tyres which required regular maintenance to fix punctures created problems for users. Tubeless tyres were preferred in the prevalent rough terrains of both rural and urban settings:

‘… I like playing with my friends but my tyres usually give me a problem because they puncture easily. Yes, our play areas are not good for inflatable tyres; maybe tubeless ones will help…’ (Male, 9, user)

### Function with wheelchair

Pre-test ratings show that between 41.8% and 74% of participants felt their previous wheelchair contributed to function, independence and mobility (Table 5). The proportion of satisfied users improved to more than 75% through implementation of the CMSP. This improvement was statistically significant in all categories (*p* ≤ 0.005) with a small confidence interval range (Table 5). The biggest improvement was shown in comfort needs (44.3%); indoor mobility (43.2%); outdoor mobility (37.2%); safe, efficient, independent operation (33.5%); and transport (31.4%).

**TABLE 5:** Comparison of ‘Functioning Every day with a Wheelchair Questionnaire’ ratings before and after CMSP services (*n* = 55)

Function item	Percentage of participants who agree that wheelchair size, fit, postural support and functional features…				Not applicable	
	
	Before	After	95% CI	*P*	Before	After
…contribute to carrying out daily routines	64.8	94.3	-67.1 to -36.9	0.001	14.8	1.9
…match their comfort needs	53.7	98.0	-77.8 to -50.2	0.001	9.3	-
…match their health needs	74.0	87.0	-51.4 to -18.6	0.001	5.6	11.0
…allow safe and efficient independent operation	61.0	94.5	-68.9 to -37.1	0.001	17.0	13.5
…allow reach and carrying out tasks at different surface heights	64.8	88.6	-60.1 to -25.9	0.001	18.5	9.4
…allow transfers	64.8	75.9	-45.0 to -9.0	0.005	20.4	16.7
…allow carrying out personal care tasks	63.0	81.5	-52.5 to -17.5	0.001	20.3	14.8
…allow getting around indoors	41.8	85.0	-62.7 to -29.3	0.001	20.0	11.3
…allow getting around outdoors	42.0	79.2	-68.9 to -37.1	0.001	17.0	15.1
…allow use of personal or public transportation	43.6	75.0	-55.5 to -20.5	0.001	21.8	7.7

*Source*: From authors own work

User feedback from the qualitative data emphasised participants’ satisfaction with function in their wheelchairs. Users reported improved independence, integration and participation, and many felt that they were now contributing to household activities rather than being a burden:

‘I can go play outside… that’s why I like this one…’ (Male, 9, user)

‘I am very satisfied when I do my work in my wheelchair without asking for too much assistance. The thing is, I don`t want to be seen as using people to do my work just because of my disability…’ (Male, 27, user)

‘I am very happy I can go where I want and can play with my friends in my wheelchair.’ (Male, 9, user)

‘…when it helps me do what I want to do and go where I have to go…it’s like I am no longer a burden and that’s what I prefer…’ (Female, 33, user)

‘My child is now able to do most things on her own including assisting with sweeping the house.’ (Female, 35, caregiver)

## Discussion

### Satisfaction with wheelchair features and function

**FIGURE 2:**
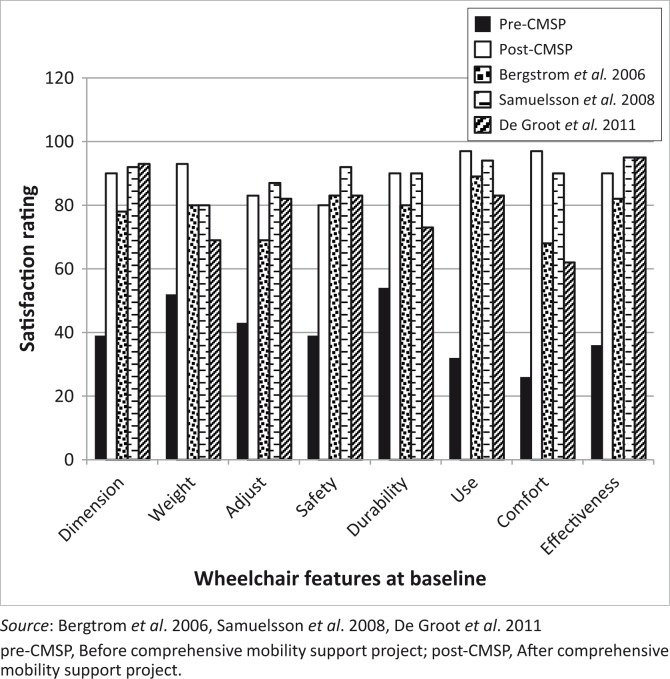
Comparing adult QUEST 2.0 satisfaction ratings with wheelchair features at baseline pre-CMSP, after implementation of the post-CMSP and other studies.

A marked improvement between pre- and post-test scores in both satisfaction with wheelchair features and function were seen. The adult post-test satisfaction ratings with wheelchair features were similar to findings from resourced settings (Figure 2) (Bergstrom & Samuelson 2006; De Groot *et al.* 2011; Samuelsson & Wressle 2008).

Significant improvement in indoor and outdoor mobility was reported (Table 5). Pre-test ratings demonstrated a large difference between satisfaction with performing functional tasks and indoor and outdoor mobility. Post-test indoor mobility ratings are on par with functional tasks while outdoor mobility was slightly lower (Table 5). As demonstrated by Visagie *et al.* (2015), any wheelchair will facilitate independence and the ability to do functional tasks. However, if the wheelchair does not match the environmental needs, satisfaction with mobility is lower than for function (Visagie *et al.* 2015). Appropriate wheelchairs that match both functional and environmental needs led to a significant improvement in mobility and satisfaction with task performance. Furthermore, satisfaction with safe and independent operation also improved significantly (Table 5). Key features such as adjustable centre of mass and rear wheel settings together with a longer wheelbase improved mobility, safety and function, particularly over rough terrain (Karmarkar *et al.* 2009; Medola *et al.* 2014; Rispin & Wee 2015). Three of the wheelchairs were semi-lightweight (16 kg), which could have further contributed to improved function and mobility (Karmarkar *et al.* 2009). All wheelchairs were available in a range of sizes which do not only facilitate fit, comfort and posture support but also allowed improved function and independence. Comfort together with ease of use had been associated with a significant improvement in satisfaction (Karmarkar *et al.* 2009).

Satisfaction with wheelchair features was strongly associated with satisfaction in function and participation in the qualitative data. Users who were satisfied with their function, independence and participation in life roles were also satisfied with the wheelchair. Similarly, other authors have found that satisfaction with wheelchair features is associated with improved participation (De Groot *et al.* 2011) and quality of life (Chan & Chan 2007), and that an inappropriate wheelchair can limit participation more than the impairment and/or the environment (Chaves *et al.* 2004).

Some features reportedly had a negative impact on function. The non-folding designs and the bulky components of folding rigid frames often resulted in transport challenges. In a South African study which reported similar transport challenges (Visagie, Duffield & Unger 2015), further research in wheelchair design is advocated to improve foldability but maintain the benefits of a rigid design such as reduced weight and improved durability and ergonomics for mobility and stability. The second challenge was related to pneumatic rear tyres where punctures limited function and participation. The increased cost of flat-free solutions may be outweighed by the gains in independence, satisfaction and participation.

All semi-lightweight wheelchairs provided through the CMSP were rigid frame wheelchairs. The only available folding frame active wheelchair was relatively heavy and available in four adult sizes only. There was no suitable alternative other than the basic folding frame for users who needed an active wheelchair with posture support options such as a higher backrest and armrests. The availability of an adjustable folding frame wheelchair would have effectively filled this gap.

Adult users reported a large improvement in comfort with the proportion of satisfied participants increasing from 26% to 96.7% (Table 3). Comfort in this study was also rated higher than in studies from resourced settings (Figure 2). This might be because users in the current study after having mostly used wheelchairs with no adjustable posture support features have now for the first time received a wheelchair which fitted well, and was tailored to their posture support needs through the multiple posture support features of the available wheelchairs. Their previous wheelchairs were often only available in limited sizes, had little posture support options or adjustments and were provided with no or fragmented accompanying services (Visagie *et al.* 2015). It is unlikely that wheelchair users in European countries ever experienced the discomfort of these limitations in their wheelchairs and services.

### Satisfaction with Wheelchair services

**FIGURE 3:**
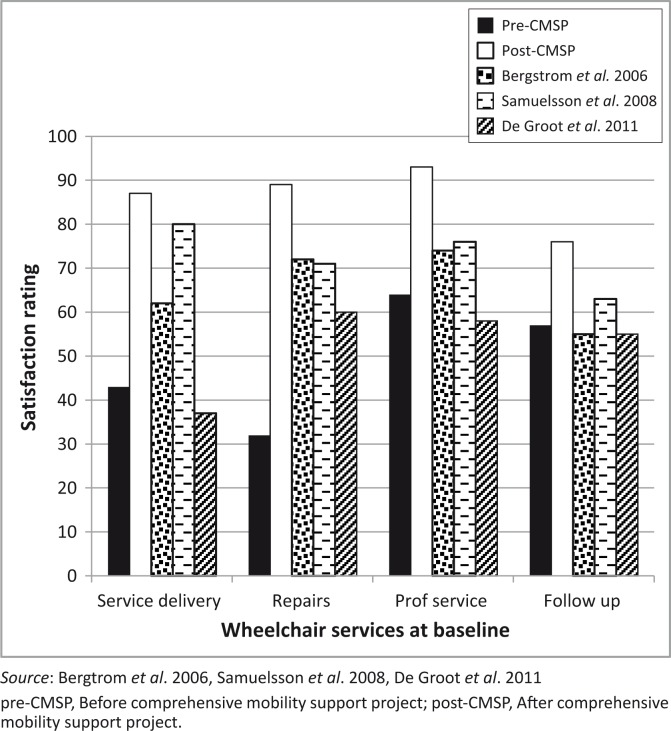
Comparing adult QUEST 2.0 satisfaction ratings with wheelchair services at baseline pre-CMSP, after implementation of the post-CMSP and other studies.

In the current study, post-test satisfaction ratings for wheelchair services surpassed that of studies in resourced settings (Figure 3). Rather than the services being exceptional, this is probably more a reflection of the immediate impact of structured services after a previous void. Key service elements on which users reflected positively in the focus groups included having a service available, short waiting periods, timeliness, trained staff, a user-centred approach and user training. These factors ultimately culminated in ‘a chair made for me’.

All clinical wheelchair service providers in this study were trained in basic and intermediate wheelchair service delivery. Wheelchair users commented positively on provider knowledge and associated this with improved satisfaction with wheelchair services. Service providers in this study were primarily rehabilitation technicians, thus confirming that wheelchair service delivery is more dependent on appropriately trained staff than a specific category of staff (UN 2006; WHO 2008).

Despite external funding support, service delivery was not without problems and/or limitations. Shortcomings in available wheelchair design and technology was discussed before. Follow-up was also flagged as a problem by users and service providers, particularly for those users from more remote areas because of distances, road infrastructure and transport challenges. These three challenges are not unique to the study setting and a very real concern in large parts of Africa (Porter 2014). While not statistically significant, the proportion of adult users that were satisfied with follow-up increased from 57% to 76%. The lack of statistical significance might be because the proportion of adult users who were satisfied by pre-test follow-up services was already above 50%. However, it is more likely that because of the short time period between receiving the wheelchair and post-test data collection (3 to 6 months), participants had not needed follow-up services other than for purposes of the study.

As this project received specific funding, sustainability of the services in the current economic context is a concern and one which was flagged by users. According to Riddel (2014), initial success of foreign aid projects does not necessarily translate into ongoing success, as sustainability challenges can develop over time, unless projects strengthen public institutions and encourage the retention of skilled staff. The CMSP was based on partnerships between the existing service providers, the government and non-government organisations (NGOs). Existing service providers received ongoing training and capacity building over an extended period to ensure adequate time for mentoring and support. Although losing skilled staff always remains a risk, the high number of rehabilitation technicians trained might contribute to a stable, skilled workforce as this cadre has limited options for employment outside their current employment sector and country. It is expected that donations will remain the main vehicle for obtaining wheelchairs, and sustainability of wheelchair services will therefore be dependent on appropriate management, coordination and distribution of wheelchair donations. The main concern about sustainability remains the funding of spares, materials and consumables. Without these items, follow-up, repair and maintenance services, as well as provision of posture support devices, will be severely hampered.

## Limitations

The following limitations must be kept in mind when interpreting and using study results. The study sample was small (*n* = 55). The focus groups included users and service providers which might have inhibited honest participation from either or both groups. Some of the data collectors were also service providers at the seating clinics. Even though the data collectors and the standard participant information sheet (translated into the two vernacular languages) emphasised that neither refusal nor honest opinions would negatively influence service provision, it could have caused bias as participants may have wanted to please service providers with their answers in order to gain favour and future services. Furthermore the existing wheelchairs which were scored during pre-test evaluation might have been old or even broken and not in the same condition as when issued new. Thus, these wheelchairs might have been compared unfavourably with the new wheelchairs of the post-test. Users might not have been able to recall their initial satisfaction with and function in the pre-test wheelchair. Users might also have struggled to recall their satisfaction with services provided a long time ago. Based on the manufacturer warrantees of the majority of the wheelchairs issued, the lifespan of the wheelchairs should exceed 3 years. Thus, assessing durability after 3 months will not provide a true reflection of the durability of the wheelchairs.

Reliability and validity of the measuring instruments in the study context were not assessed. Context and culture can influence the way in which people understand and respond to questions. It can also influence user’s views on the relative importance of variables. Thus, variables important to the current study population might not have been assessed by the tools.

## Conclusion

The post-test QUEST and FEW ratings following the implementation of the CMSP demonstrate statistically significant improvement in satisfaction with all except two categories (adult satisfaction with follow-up and child satisfaction with training) of wheelchair features, service delivery and function. The study findings also illustrate that wheelchair users in low-resource settings can experience similar satisfaction levels with wheelchairs, services and function as wheelchair users from resourced settings, despite fewer resources and using more basic technology. Since the CMSP was based on the WHO wheelchair guidelines (WHO 2008), one might also conclude that service provision in accordance with these guidelines does result in satisfactory wheelchair services and improved user function in less resourced settings. This study further demonstrates that problems with sustainability, particularly related to funding and service providers, might be expected.

## Recommendations

It is recommended that the Zimbabwean government together with the current partner organisations continue to support and further develop wheelchair services by specific policy, management and service guidelines, which could include a coordinated management approach for wheelchair donations. Since sustainability is also dependent on trained personnel, the ongoing training and capacity building of existing service staff should be a key feature, together with integration of the WHO WSTP-B into the training curriculum of service providers. Finally, as implementation of the WHO wheelchair guidelines seems feasible, services in other low-resource settings should also implement and research the impact of the guidelines.

## Footnotes

1 Occupational therapists, physiotherapists, orthopaedic technologists, orthotist/prosthetists and rehabilitation technicians.

2 The WHO WSTPs are based on evidence and international best practice guidelines and focus on universal skills and standards that are achievable within all service settings.

3 Wheelchair technologists and technicians, orthotists/prosthetists and welders.

4 Folding rigid frame refers to a wheelchair with a rigid frame design of which some components are removable or can fold, for example, quick-release rear wheels or a fold-down back rest. This allows the wheelchair to be broken down into smaller components for easier transport or storage. In contrast, a non-folding rigid frame has no removable or foldable components and the wheelchair must be transported as a complete unit.
